# A Comparative Analysis of Data Analysis Tools for Data-Independent Acquisition Mass Spectrometry

**DOI:** 10.1016/j.mcpro.2023.100623

**Published:** 2023-07-21

**Authors:** Fangfei Zhang, Weigang Ge, Lingling Huang, Dan Li, Lijuan Liu, Zhen Dong, Luang Xu, Xuan Ding, Cheng Zhang, Yingying Sun, Jun A, Jinlong Gao, Tiannan Guo

**Affiliations:** 1 Center for Intelligent Proteomics, Westlake Laboratory of Life Sciences and Biomedicine, Hangzhou, Zhejiang Province, China; 2 Key Laboratory of Structural Biology of Zhejiang Province, School of Life Sciences, Westlake University, Hangzhou, Zhejiang Province, China; 3 Westlake Omics, Ltd, Hangzhou, Zhejiang Province, China

**Keywords:** data-independent acquisition, EncyclopeDIA, DIA-NN, OpenSWATH, Skyline, Spectronaut, mass spectrometry, proteomics

## Abstract

Data-independent acquisition (DIA) mass spectrometry–based proteomics generates reproducible proteome data. The complex processing of the DIA data has led to the development of multiple data analysis tools. In this study, we assessed the performance of five tools (OpenSWATH, EncyclopeDIA, Skyline, DIA-NN, and Spectronaut) using six DIA datasets obtained from TripleTOF, Orbitrap, and TimsTOF Pro instruments. By comparing identification and quantification metrics and examining shared and unique cross-tool identifications, we evaluated both library-based and library-free approaches. Our findings indicate that library-free approaches outperformed library-based methods when the spectral library had limited comprehensiveness. However, our results also suggest that constructing a comprehensive library still offers benefits for most DIA analyses. This study provides comprehensive guidance for DIA data analysis tools, benefiting both experienced and novice users of DIA-mass spectrometry technology.

## Highlights

• Reproducible data-independent acquisition proteome data analysis workflows.
• Library-based *versus* library-free approaches for DIA searches.
• Comparison of identification and quantification metrics.
• Guidance for DIA data analysis.

## Graphical Abstract

**Figure:**
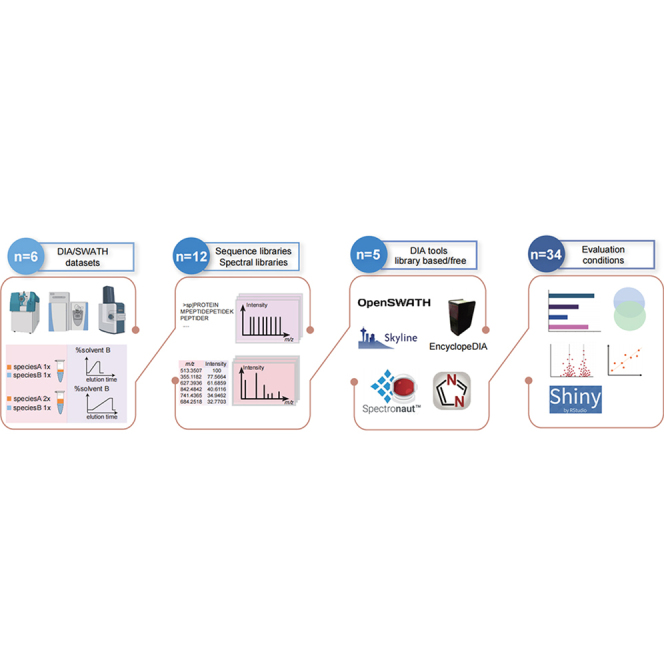

## In Brief

A guideline study for choosing DIA software tools for both experienced and novice users. Evaluation of 5 DIA tools (OpenSWATH, EncyclopeDIA, Skyline, DIA-NN, Spectronaut) in 6 different data sets, with 12 libraries for 34 conditions. The library-free approach excels when the spectral library size is limited, yet building a comprehensive library remains advantageous for most DIA analyses.

Complex specimens can be profiled for thousands of proteins through the use of tandem mass spectrometry (MS)-based proteomics (1, 2). In contrast to the traditional data-dependent acquisition (DDA) method that selectively chooses peptides for fragmentation, data-independent acquisition (DIA) is a MS approach that involves fragmenting all ions within a specific *m/z* range (3, 4, 5). DIA-MS offers a comparable level of reproducibility to targeted proteomics; however, it is not limited to a specific set of peptides. The reproducibility of DIA-MS has been well established in a few cross-laboratory studies (6, 7). The high reproducibility of DIA-MS serves as a crucial foundation for acquiring high-throughput proteome data from large-scale clinical sample cohorts, particularly in the context of training machine learning–based models. This reproducibility ensures consistent and reliable data acquisition, enabling the generation of robust and accurate models that can effectively analyze and interpret the complex proteomic information obtained from clinical samples.

In DIA-MS, a significant challenge lies in the deconvolution of multiplexed fragment ion spectra, as the relationship between precursor and fragment ions is lost. To address this challenge, two main approaches are commonly employed for identifying and quantifying peptide precursors in DIA data: library-based and library-free methods (8). Library-based method is the first widely used strategy in DIA analysis which analyzed DIA data using a preconstructed spectral library comprising of the relative intensity of peptide fragment ions and retention time (RT), which can be generated from fractionated DDA data (9, 10), or predicted from precursor sequences (11, 12, 13). The library-based analysis tools improved performance on the algorithms in matching MS signal with spectral libraries, scoring features construction, and statistical modeling for false discovery rate (FDR) estimation. Library-free approaches do not require prebuilt libraries but uses a protein sequence database or predicted libraries to analyze DIA-MS data (14). While library-free methods offer certain advantages, such as flexibility in analyzing DIA data without relying on preconstructed spectral libraries, they often require additional inspection for controlling FDRs. DIA-MS has primarily been adapted for three types of MS machines: Sciex’s TripleTOF, Thermo’s Orbitrap, and Bruker’s TimsTOF Pro. The main differences between these mass analyzers lie in their resolutions and the specific implementations of DIA methods. As a result, different software tools (DIA-NN (https://github.com/vdemichev/DiaNN) (11), EncyclopeDIA (https://bitbucket.org/searleb/encyclopedia) (15), OpenSWATH (http://openswath.org) (16), Skyline (https://skyline.ms) (17), and Spectronaut (https://biognosys.com/software/spectronaut) (18)) have been optimized to cater to either library-based or library-free DIA analyses, specifically tailored for different vendors' instruments. This ensures that the software tools are compatible and optimized for the specific characteristics and data generated by each type of MS machine.

Earlier comparisons of DIA data analysis tools were conducted (19) at a time when the DIA method was just beginning to gain widespread use. However, these comparisons were limited to a small number of datasets and MS instruments. The past years have witnessed the rapid evolution of DIA methods for various mass spectrometers and, consequently, of new and more advanced DIA data analysis tools. A recent study (20) compared the performance of several DIA data analysis tools including DIA-NN, DIA-Umpire, OpenSWATH, ScaffoldDIA, Skyline, and Spectronaut. However, it is worth noting that this study was limited to artificially simplified samples containing only 48 human proteins and focused solely on Orbitrap-based machines. Despite these limitations, the study found that these tools, despite their distinct implementations, did not generate significantly divergent identifications and quantifications of peptides or proteins. Nevertheless, there is a need for systematic characterization of the differences and consistencies between the current DIA data analysis tools. Such information would be beneficial for both DIA data analysis software developers and researchers, utilizing DIA in their biological studies. In particular, a cross-tool comparison can assist users in selecting the most suitable tool for their specific research questions and requirements.

Here, we evaluated five widely used DIA data analysis tools: OpenSWATH, EncyclopeDIA, Skyline, DIA-NN, and Spectronaut. Our analysis involved six DIA datasets generated from three different types of mass analyzers (Triple TOF, Orbitrap, and TimsTOF Pro). We employed two approaches for empirical true positive validations and conducted searches using both library-based and library-free modes. Throughout the study, we examined the consistencies and discrepancies among the assessed tools in terms of identification and quantification. We further investigated the factors contributing to these findings. Based on our results, we provided a comprehensive guide to aid researchers in selecting and effectively utilizing different DIA data analysis tools. To facilitate DIA data analysis, we also developed a freely accessible web server that consolidates the search results produced by various DIA data analysis tools. This server offers a user-friendly interface and can be accessed at https://www.guomics.com/softw/diatoolcomp.

## Experimental Procedures

### Spectral Library Conversion

**Fig. 2:**
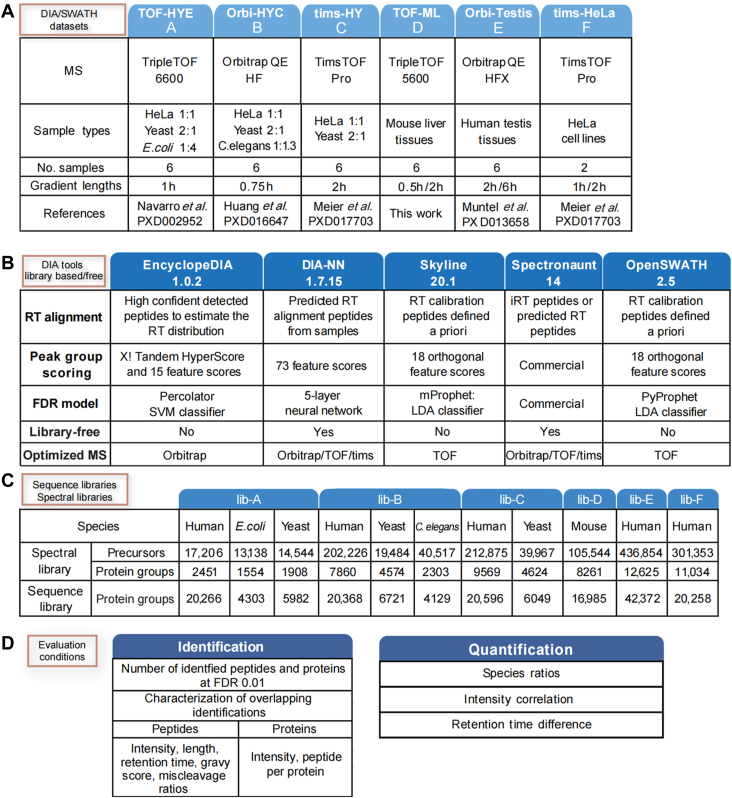
**Study details.***A*, details of the DIA datasets were used to evaluate the data analysis tools. *B*, for each dataset, the composition of their spectral and sequence libraries is detailed here. *C*, three main aspects enclose the most relevant features of the DIA data analysis tools that we evaluated: RT alignment, peak group scoring, and the FDR model. *D*, details of the metrics used to evaluate the identification and quantification results. DIA, data-independent acquisition; FDR, false discovery rate; RT, retention time.

Raw spectral libraries and FASTA sequence libraries from selected studies (4, 9, 21, 22, 23) were downloaded. Specifically, lib-A, lib-B, lib-C, and lib-E (Fig. 2*B*) were downloaded from PXD002952, PXD016647, PXD017703, and PXD013658 as Spectronaut xls files. lib-D, also in xls format, was built using Spectronaut. Lastly, lib-A was downloaded in OpenSWATH tsv format. While DIA-NN can read Spectronaut xls files without further conversion, we wrote an in-house R script to convert the xls libraries into a format suitable for OpenSWATH. Specifically, the modification expression was changed from brackets into UniMod format. Also, the mappings were changed from "[carbamidomethyl (C)]" to "(UniMod:4)," from "[oxidation (M)]" to "(UniMod:35)," and from "[acetyl (protein N-term)]" to "(UniMod:1)." Finally, the file columns were mapped into the corresponding columns of the OpenSWATH tsv file. TargetedFileConverter in OpenSWATH was then used to convert the OpenSWATH tsv file into the OpenSWATH TraML format. The TraML files were imported into EncyclopeDIA in dlib format. Lastly, the blib file format, which can be imported into Skyline, was generated using EncyclopeDIA.

### Parameters for the Five DIA Tools

This section summarizes the parameters used for each DIA data analysis tool. EncyclopeDIA was used in the target-decoy mode, with equal numbers of target and decoy peptides. The decoys were generated in reverse mode. The precursor, fragment, and library mass tolerances were set as 10 ppm for the Orbitrap datasets and 25 ppm for the SWATH datasets. Percolator 3.0.1 (https://github.com/percolator/percolator/) was used to estimate the peptide and protein FDRs. The decoy peptides for OpenSWATH were generated using OpenSwathDecoyGenerator, and the results were converted into PQP file using TargetedFileConverter. PyProphet was used to generate the peptide identification result with peptide and protein FDR control. Detailed command lines are provided in supplemental Fig. S5. Spectronaut was used with the default factory settings provided in the user interface, as detailed in supplemental Fig. S4.

The source-specific indexed retention time (iRT) calibration was allowed, and the calibration mode was set as “automatic.” The *m/z* extraction strategy was set as “maximum intensity.” The mass tolerances of both MS1 and MS2 were set as “system default strategy.” Other options selected include “precision iRT,” “exclude deamidated peptides,” and “iRT regression by local nonlinear regression”. The decoy for spectral library was generated using the “mutated” option with the decoy limit strategy set as “dynamic;” the “library size fraction” was set as 0.1 with the “machine learning” option set as “per run.” “posttranslational modification localization” was selected with a probability cut-off of 0.75. For the quantification, we used the Top-6 transition ion at MS2 level. “Cross-run normalization” was selected and set to use the “global normalization strategy.” The “proteotypic filter” was set as “none”. The major grouping was performed by “protein group ID,” while the minor peptide grouping by “stripped sequence.” The major group was set as “mean peptide quantity,” and “major group Top1-3” was selected.

For the diaPASEF dataset in Spectronaut, the extraction of the ion chromatograms and ion mobility window was set as “dynamic” with correction factor 1. Other settings were the same as for the DIA analysis. For the library-free search, the factory settings were used. The length of peptides was set to range between 7 and 52 amino acids, with a maximum of five variable modifications and two missed cleavages. Other parameters were the same as for the library search.

For DIA-NN, the dynamic correction of mass accuracy was used. In addition, the options “process in batches,” “use neural network,” “use isotopologues,” “RT profiling,” and “remove likely interference” were chosen. FDR cut-off was set as 0.01 at the precursor level.

For Skyline, the decoy peptides were appended in reverse mode. For peptide identifications, we generated a peak scoring model with mProphet using scores derived from intensity, RT difference, library dot-product, weighted shape, weighted co-elution, co-elution count, signal to noise, and product mass error.

### Compilation of a Comparative Report for Each Data Analysis Tool

Each data analysis tool generated results in different formats. To enable comparisons between different tools, we generated a unified data format. We used the mean values when a peptide included multiple charge states. To avoid the complexity of the modified peptides, we only considered the unmodified peptides.

For EncylcopeDIA, the peptide (elib.peptides.txt) and protein (elib.proteins.txt) quantification matrices were reported as result files without precursor files. In addition, the peptide modifications were changed into UniMod format.

For DIA-NN, peptide precursors (pr.matrix) and protein matrices (pg.matrix) were used as the result files. The mean intensity of a peptide’s precursors with an identical stripped sequence was used as the peptide’s intensity.

For OpenSWATH, the precursor peptide matrices were reported as the output. The columns FullPeptideName, charge, and intensity were extracted to form the precursor matrix. The peptide intensity matrices were generated using the mean intensity of the peptide precursors sharing identical stripped sequences. Protein intensity was computed from the three most abundant precursors of each protein using ProteomeExpert (24).

For Skyline, Total.Area and Precursor.Charge were used as the skyline result files to generate the precursors’ intensity matrices. Peptides and proteins’ intensity matrices were computed similarly to OpenSWATH. Additionally, the modifications were changed to UniMod formats.

Finally, for Spectronaut, PEP.Quantity, PG.ProteinGroups, and EG.PrecursorId were chosen to report the peptide precursors, protein groups, and peptide matrices.

### Protein Identification

To avoid the variations caused by peptide modification, we only counted the number of stripped peptides without modifications. Also, to prevent the complication arising from the protein groups, we only used the unique proteins that do not belong to any protein group in reporting the protein identification number. For the label-free quantification (LFQ) samples, the true ratios are 1:1, 2:1, and 1:4 for human, yeast, and *Escherichia coli.* in dataset A; 1:1, 1:1.3, and 2:1 for human, *Caenorhabditis elegans*, and yeast in dataset B; and 1:1 and 1:3 for human and yeast in dataset C for each pair of sample sets. The ratios were calculated using the mean values of each sample. The identifications were considered true if the experimental ratios were within 30% of the true ratios.

### UpSet Analysis

As we compared more than five data analysis tools and search strategy combinations, we used UpSet plots to visualize the intersection of the identified peptides and proteins. Every possible intersection was represented using heatmaps with their ranked occurrence shown in the left-hand bar plots. Since the number of combinations was very large, we have restricted to the top 90% cumulative occurrences for visualizing the intersections.

### Properties of Peptides and Proteins

Four peptide properties and three protein ones were used to characterize the different intersection portions further. The quantified peptide and protein intensity was individually derived from each software tool’s peptide and protein quantification matrices. A peptide’s length was counted using the number of characters (*i.e.,* amino acids) within its sequence. GRAVY values were calculated by summing the hydrophobicity values derived from the Kyte-Doolittle scale: 1.8, −4.5, −3.5, −3.5, 2.5, −3.5, −3.5, −0.4, −3.2, 4.5, 3.8, −3.9, 1.9, 2.8, −1.6, −0.8, −0.7, −0.9, −1.3, and 4.2 for A, R, N, D, C, Q, E, G, H, I, L, K, M, F, P, S, T, W, Y, and V, respectively (25). Missed cleavages were counted through the numbers of K and R within a peptide’s sequence minus one. The RTs of the peptides were matched from the normalized RT reported in the library. The molecular weights of the proteins were calculated by summing the monoisotopic molecular masses of their amino acids according to their full FASTA-file sequences. The numbers of peptides per protein were computed based on the source protein of each identified peptide.

### Coefficients of Variance

CVs were calculated using SDs divided by the mean values of the precursors, peptides, and proteins in a replicate set. The CV values were calculated for precursor, peptide, and protein intensity.

### LFQ Analysis

**Fig. 6:**
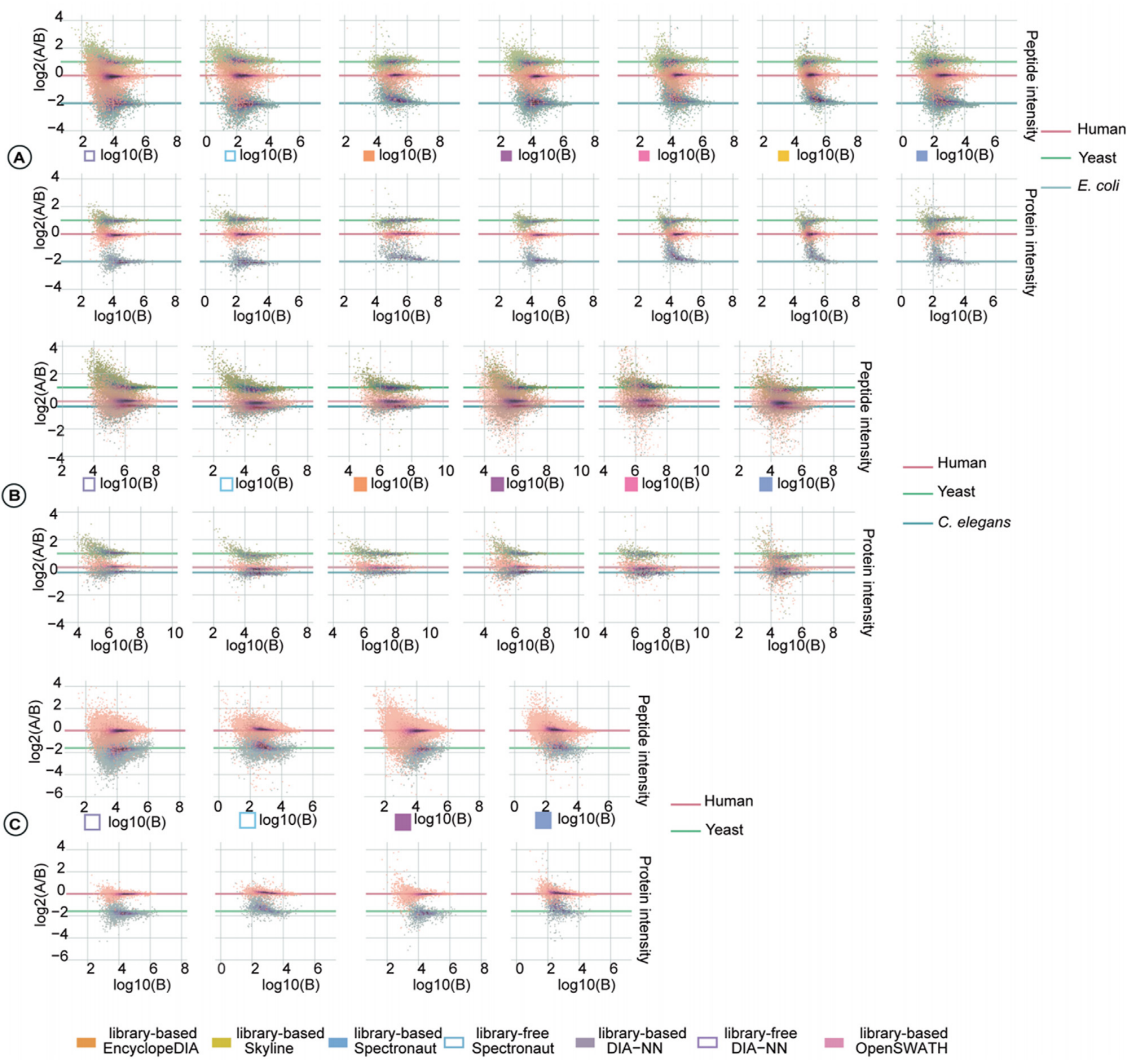
**Evaluation of quantification in multispecies datasets.** The plots show the distributions of the log2(*A* and *B*) against log10(*B*) of the intensity values for datasets *A*–*C* at peptide and protein levels. The tools are indicated with *color legend* in the *middle*. The species names are indicated at the *right* for each dataset. *Solid lines* indicate the true quantification ratios.

We plotted the log2 ratio of the intensity of the true positive peptides or proteins of sample A *versus* sample B, log2(A/B), against the log10 intensity of sample B for each species (supplemental Fig. S9). Combined violin plots show the distribution of the LFQ ratios in Figure 6. The left-hand plots show the distribution of the log2(A/B), whereas the right-hand plots show the normalized distribution of log10(B). The normalization was calibrated to each data analysis tool's minimal and maximal intensity.

### Cross-Tool Correlation of Intensity and RT

To perform a pairwise comparison of the data analysis tools, we computed Pearson’s correlations between the intensity of matching peptides and proteins as returned by different tools. In addition, Pearson’s correlation of the RT apexes was also calculated, for each tools pair, using matching peptides. Finally, the distribution of the RT differences was plotted using violin plots and geom_violin in ggplot2, with variable windows sizes to fit each dataset.

## Results

### Study Design

**Fig. 1:**
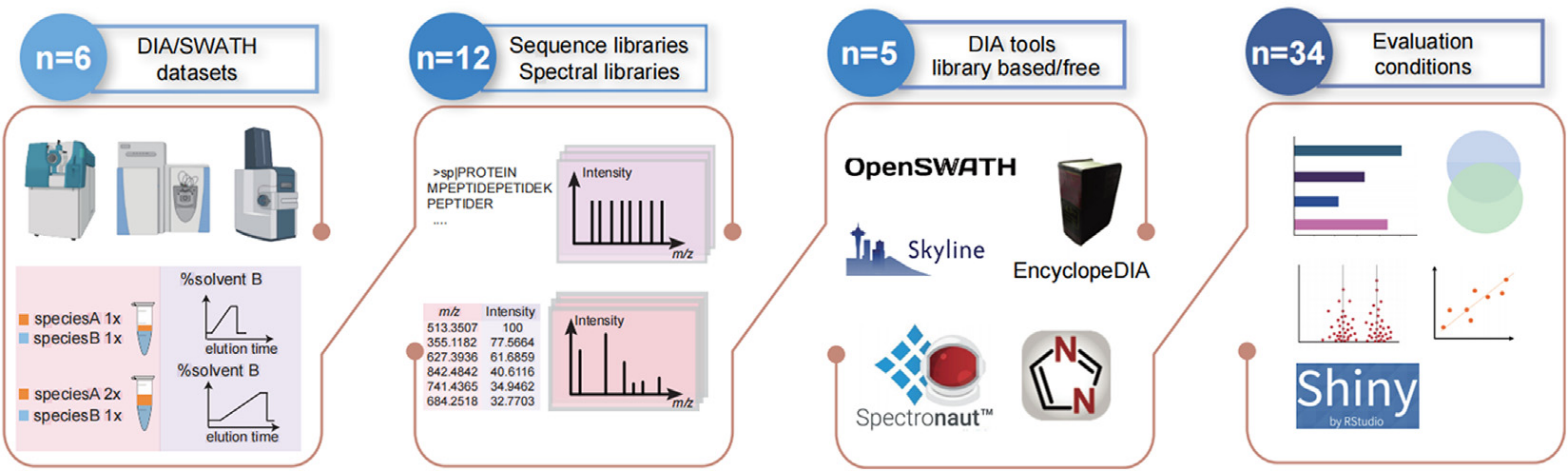
**Study workflow.** The study workflow involves the utilization of six datasets generated from three types of mass spectrometers. These datasets are used to benchmark five DIA data analysis tools: OpenSWATH, EncylcopeDIA, Skyline, Spectronaut, and DIA-NN. For each dataset, a total of 12 libraries, consisting of six sequence libraries and six spectral libraries, are analyzed using the five DIA data analysis tools. The performance evaluation of the data analysis tools comprises 34 different tests. The tests assess various aspects, including the numbers of identified peptides/proteins, overlaps between identifications, CVs, and cross-tool correlations. An R-shiny server is developed. DIA, data-independent acquisition.

The workflow of our comparative study of DIA data analysis tools can be summarized in four sections (Fig. 1). Initially, we used six datasets obtained from three types of mass spectrometers, which is feasible for DIA-MS scheme. In addition to conventional target-decoy validations, we employed two additional empirical validation approaches for each mass spectrometer type.

More specifically, the multispecies datasets were derived among the first-published DIA datasets generated with each of three selected mass spectrometers. The first dataset is the classic *LFQBench* datasets (19) (*Homo sapiens*, *Saccharomyces cerevisiae*, and *E. coli*, with ratios of 1:1, 2:1, and 1:4, respectively) from TripleTOF MS instruments (dataset A: TOF-HYE). The second dataset is from three species generated by Biognosys on Orbitrap MS instruments (human, yeast, and *C. elegans*, with ratios of 1:1, 2:1, and 1:1.3, respectively) (21, 22) (dataset B: Orbi-HYC). The last dataset is a two-species data generated in diaPASEF (4) (human and yeast, with ratios of 1:1 and 2:1, respectively) (dataset C: tims-HY). For the second validation approach, we generated one in-house dataset using a TripleTOF 6600 and mouse liver samples (dataset D: TOF-ML). For the DIA Orbitrap validation, we used a dataset of testis cancer tissues that measures more than 10,000 protein groups on a Q Exactive HF-X Orbitrap (dataset E: Orbi-testis) (23). For TimsTOF Pro, we chose the HeLa cell lysates dataset from the very first diaPASEF work (dataset F: tims-HeLa) (Fig. 2*A*).

Five software tools for DIA analysis, which are still actively improved, were chosen for this comparative study: EncyclopeDIA (version 1.0.2) (15), DIA-NN (version 1.7.5) (11), OpenSWATH (version 2.5.0) (16), Skyline (version 20.1) (17), and Spectronaut (version 14.5) (18). At the beginning of the study, PEAKS was not chosen because its main emphasis was on *de novo* identification, and it lacked the capability to use MS2 ions for quantification. Nevertheless, since 2021, PEAKS has been enhanced to include functionality for DIA identification and quantification using MS2 ions. Furthermore, it now offers various approaches such as spectral library-based search, library-free search, and *de novo* search. MaxDIA (https://github.com/JurgenCox/compbio-base) (v2.4.2) was not used due to its limited computational speed and prone to technical crashes, making it unsuitable for comprehensive dataset comparisons. Through personal communications, we have been updated that newer versions of MaxDIA with significantly improved performance are being developed. Each of the evaluated tools in this study was developed and optimized for specific types of MS instruments. Figure 2*B* provides a summary of the three key algorithms where these tools exhibit the most significant differences. These algorithms encompass signal calibration for library alignment, peak group scoring based on various chromatographic or spectral features, and FDR estimation using classifiers. Only Spectronaut and DIA-NN supported library-free search mode. In this study, the analysis parameters of each software tool were set to their default values and modes, assuming that most users tend to use them as such. The specific parameters used for EncyclopeDIA, Spectronaut, OpenSWATH, DIA-NN, and Skyline are provided in the Methods section, as described in supplemental Figs. S3–S7. Overall, the selection and configuration of the tools and their parameters were designed to reflect typical user practices and enable a comprehensive evaluation of their performance on the DIA datasets.

Two library search strategies were used for each dataset, resulting in six library-based and six library-free libraries. Library-based method is the first widely used strategy in DIA analysis, which analyzes DIA data using a spectral library. The spectral library comprises the relative intensity of peptide fragment ions and RT, which can be generated from fractionated DDA data or predicted from precursor sequences. In this study, for the published datasets (datasets A, B, C, E, and F), spectral libraries were derived from the libraries provided in the original publications while the in-house dataset (dataset D) was from the associated in-house generated libraries (lib-D). Spectral lib-A contains 2451, 1554, and 1908 protein groups for human, *E. coli*., and yeast, respectively. Spectral lib-B contains 7860, 4574, and 2303 protein groups for human, yeast, and *C. elegans*, respectively. Spectral lib-C contains 9596 and 4624 of human and yeast proteins, respectively. Spectral lib-D contains 8261 mouse protein groups. Spectral lib-E contains 12,625 human protein groups, while spectral lib-F contains 11,034 human protein groups. Here, we comment on the size of libraries for the five human-associated libraries by percentage of 20,000 human encoding proteins: lib-A is considered as fairly small (around 10%); lib-B and lib-C are in middle size (30–40%); and lib-E and lib-F are considered as comprehensive libraries (∼50%). All the spectral libraries were further converted to the file formats required by each tool (supplemental Fig. S2). On the other hand, library-free approaches converted DIA spectra into pseudo DDA spectra and can be searched with FASTA sequence library like DDA data. The sequence FASTA libraries are the same FASTA file used to build the spectral library. The sizes of the sequence libraries were 5.18, 2.12, 1.88, 2.06, 3.36, and 1.84 times larger than that of the corresponding spectral libraries for lib-A, lib-B, lib-C, lib-D, lib-E, and lib-F, respectively (Fig. 2*C*).

In our evaluation, we conducted analyses on the six datasets using the five data analysis tools, both in library-based and library-free modes whenever available. However, due to limitations in processing TimsTOF data, library-free analyses could only be performed using DIA-NN and Spectronaut. In total, we performed 63 searches, out of which 51 were successful, six were not possible due to the lack of functionality for processing TimsTOF data, and six were unsuccessful due to technical issues. The feasibility of performing DIA analysis and the final reported formats (precursor, peptide, or protein matrices) is summarized in supplemental Fig. S1. The performance of the tools was compared at two basic levels: identification and quantification, as illustrated in Figure 2*D*. These comparisons allow for a comprehensive assessment of the tools' capabilities in terms of accurately identifying and quantifying peptides and proteins from the DIA datasets.

### Depth of Peptide and Protein Identification

In our evaluation, we initially assessed the performance of the five DIA data analysis tools by examining the number of peptide and protein identifications. To account for the potential variability introduced by modifications and protein groups, we compared the number of identified stripped peptide sequences and the corresponding unique proteins.

To ensure a reliable identification count at the precursor ion level, we applied a FDR cut-off of less than 0.01. Instead of generating a cumulative pseudo receiver operating characteristic plot of identification number against FDR, we focused on the absolute number of identifications meeting the FDR <0.01 cut-off. This approach allows for a straightforward comparison of the identification performance among the tools.

**Fig. 3:**
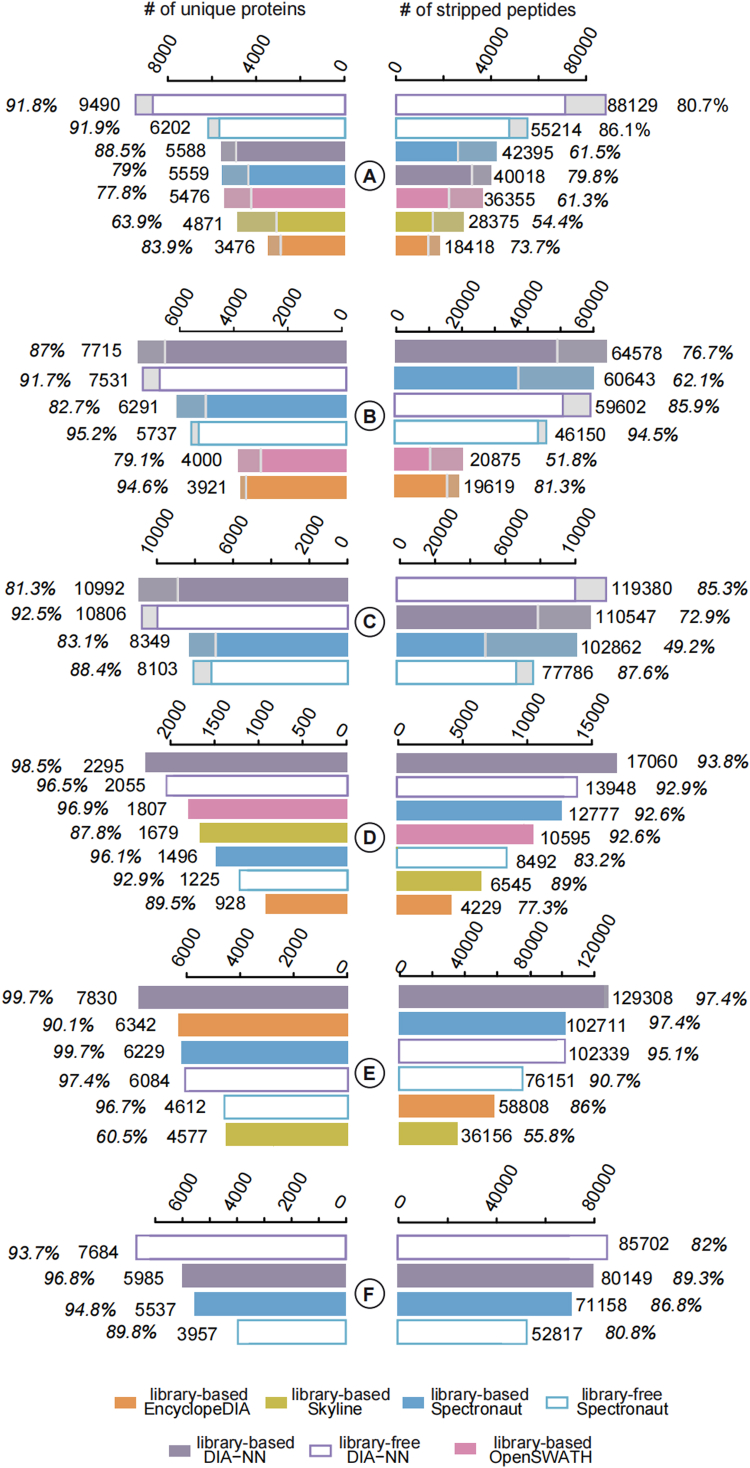
**Evaluation of peptide and protein identifications.** Stripped peptides (*top bars*) and unique proteins (*bottom bars*) are plotted for each dataset (named as *A*–*F* in Fig. 2*A*). Search modes are library-free (*hollow bars*) or library-based (*solid bars*). The *solid color region* below the *middle white line* indicates the identifications that passes the truthfulness validation.

DIA-NN significantly outperformed the other tools. Specifically, its identification numbers exceed those provided by the second-best tool (Spectronaut) by 59.6%, 6.5%, 16.1%, 33.5%, 25.9%, and 20.2% for peptide numbers and by 53.0%, 22.6%, 31.6%, 27.0%, 23.5%, and 38.8% for unique proteins, for the datasets A, B, C, E, D, and F, respectively (Fig. 3). Spectronaut surpassed the third-ranked tool by 51.9%, 190.1%, 20.6%, and 74.7% identifications for dataset A, B, D, and E, respectively. On the other hand, the number of proteins identified by Spectronaut was not the second highest for datasets D and E, possibly due to the redundancies in peptides for the same proteins. The identification performance of OpenSWATH was close to that of the two best performing tools for the two TripleTOF datasets (A and D) as it only identified 9.15% and 17.7% fewer peptides than Spectronaut. It even identified the second largest number of unique proteins in dataset D. EncyclopeDIA could not analyze the TimsTOF Pro data, and its identification performance was relatively low in the TripleTOF datasets (A and D). However, EncyclopeDIA showed a fairly good performance on the Orbitrap datasets (B and E), and it ranked second in numbers of identified proteins in dataset E. Finally, Skyline performed better on the TripleTOF datasets, achieving 12% more protein identifications in dataset D than Spectronaut.

In the field of DIA analysis, there has been a trend in discarding the time-consuming spectral library building steps as spectral libraries can be directly predicted from the sequence using deep neural network methods. In our analyses, we observed that if the size of the spectral library was much smaller (in this study 5-folds smaller) than the FASTA protein sequence library, the library-free method performed better than the library-based one. For instance, in the case of lib-A, the sequence library was 5.18 times larger than the spectral library. Using DIA-NN, the library-free methods identified 120.2% and 69.8% more peptides and proteins than the library-based methods, whereas Spectronaut achieved 30.2% and 11.6% more peptides and proteins. Additionally, DIA-NN identified 8%, 6.9% more peptides in the TimsTOF Pro dataset C and dataset F, respectively. While the number of protein identifications was 1.7% smaller for dataset C, it was 28.4% larger for dataset F. For the remaining tools, the highest number of identifications were derived again from the library-based approaches. Of note, library-free methods identified far more peptides than proteins, indicating additionally identified peptides are from already identified proteins. These peptides were “degenerated” for protein identification. For example, DIA-NN library-based approaches achieved 8.3% more peptides but only 2.4% more proteins in dataset B. Similarly, when using Spectronaut, there was a significant rise of 31.4% in peptide identifications, whereas the increment in protein identifications was only 9.7%.

The identification reliability was further assessed by the defined mixed-species quantification ratio. We included the 30% peptides or proteins above/below the true quantification ratios to determine if an identification was valid for the multispecies defined ratio datasets (datasets A, B, and C). None of the LFQ datasets reached the statistical thresholds estimated by the FDR. The library-free methods provided better accuracy than the library-based ones.

The computing time was also recorded to evaluate the computing speed of each tool (supplemental Fig. S8). Again, DIA-NN was the fastest to complete computations (though the tests were not running on the same machine, we indicated with number of cores and estimated central processing unit hours to make them comparable), followed by Spectronaut, EncyclopeDIA, OpenSWATH, and Skyline.

### Evaluation of the Cross-Tool Identifications

In order to assess the consistency and discrepancies among various tools, we analyzed the shared physicochemical properties that were deduced from the peptide or protein sequences. This analysis allowed us to assess the consistency in the identification results among the different tools. At the quantification level, we computed the quantification accuracy by analyzing the distribution of quantification ratios. This analysis provided insights into the accuracy of the quantification results generated by each tool. In addition, we conducted pairwise cross-correlation comparisons of peptide and protein intensities to evaluate the correlation between the quantification values acquired from distinct tools. By conducting these evaluations, we gained a better understanding of the similarities and differences in the identification and quantification results among the analyzed DIA data analysis tools.

**Fig. 4:**
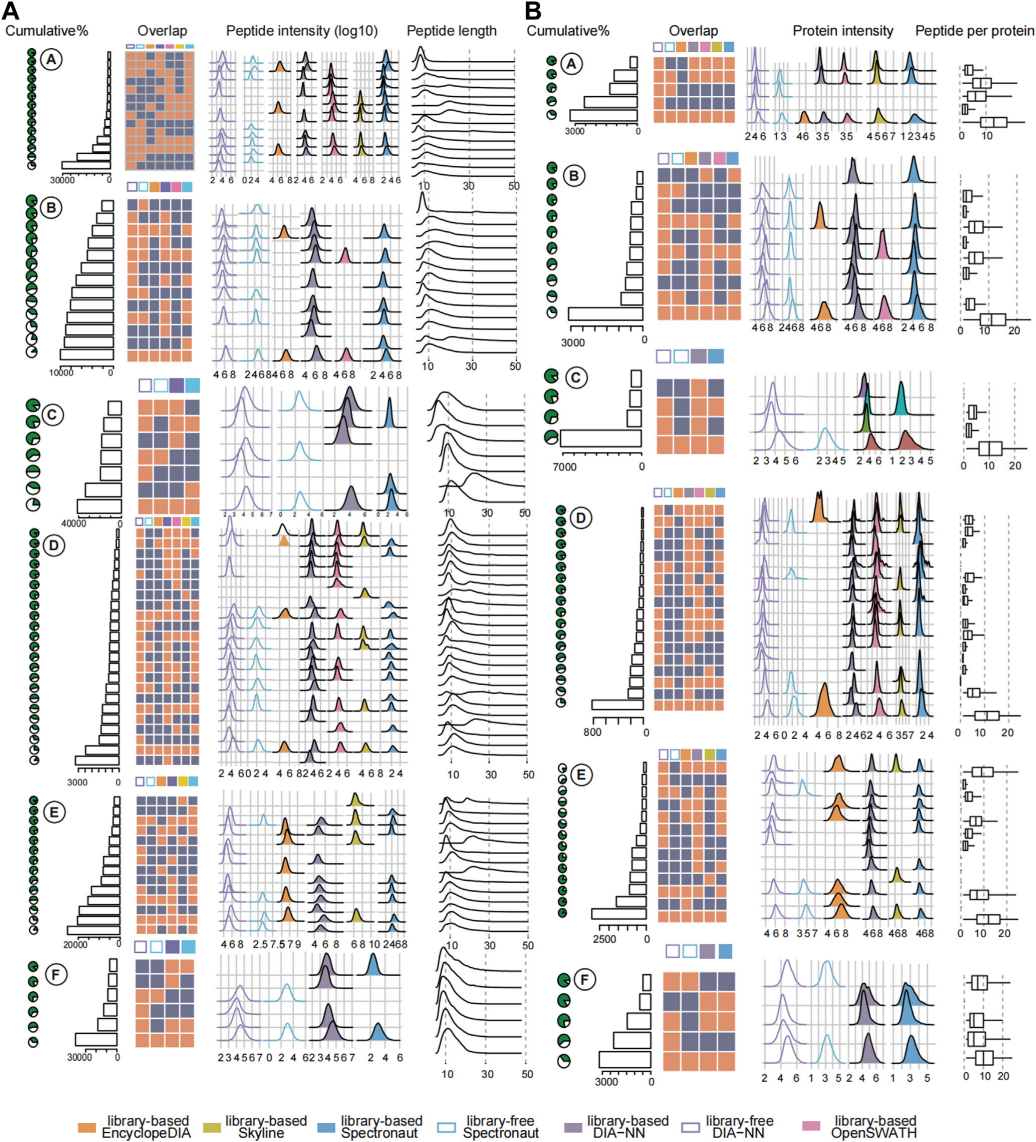
**Characterization of cross-tool identifications.** *A*, identified peptides. *B*, identified proteins. From *top to bottom*, the results from datasets *A*–*F* are shown. The “overlap” column highlights in *red* when overlapping identifications across tools are found. The *rows* describe the intersecting combinations. The cumulative count percentage is indicated in the *leftmost pie charts*. The *ridge plots* represent the distribution of the log10 peptide/protein intensity derived from different tools. Peptide lengths are indicated for peptides and peptides per proteins are indicated for proteins.

To study the source of the variable identifications across tools, we next evaluated the peptides and proteins uniquely identified by specific tools (Fig. 4*A*). As the number of combinatorial overlapping would be exploding, we only characterized the intersections that cumulatively summed up to 90% of total identifications. We found a clear positive correlation between the abundance and the number of identified tools. The peptides or proteins identified by several tools had the highest intensity, whereas those identified by fewer tools had lower intensity. In other words, the highest abundant proteins generated peptides that were more easily identified by any tool. On the contrary, peptides uniquely identified by single tools were derived from less-abundant proteins with lower signal level. Such was the case of the following analyses: library-free DIA-NN and Spectronaut on datasets A and E, library-based Spectronaut and EncylcopeDIA on dataset B, library-based Spectronaut on dataset C, library-based and library-free DIA-NN on dataset D, and library-based DIA-NN on dataset E and F.

Since more than 50% of the peptides identified by all the tools in dataset A required library-free methods, we inspected the physicochemical properties of the peptides from these portions of additionally identified by library-free methods. These peptides were shorter and with relatively low missed-cleavage ratios. Also, as they eluted during the last part of the chromatographic gradient with higher GRAVY values, they were more hydrophobic. Similar observations could be made from the library-free portion of dataset F (supplemental Fig. S9).

Four out of six datasets for the peptides (Fig. 4*A*) and all the datasets for the proteins (Fig. 4*B*) showed the highest numbers of intersections across all tools. This showed high consistency in the cross-tool DIA identifications. Furthermore, protein identifications were more consistent than peptide ones. In datasets A and D, the proteins intersecting across all the tools were of the highest identification number among all the combinatorial intersecting set. This indicates that these additionally identified peptides are redundant for protein identification improvement as those proteins are redundantly identified through several of their peptides. This is consistent with the number of peptides per protein identified, as the intersecting portion across all the tools had more peptides per protein.

### Highly Consistent Quantification Results

**Fig. 5:**
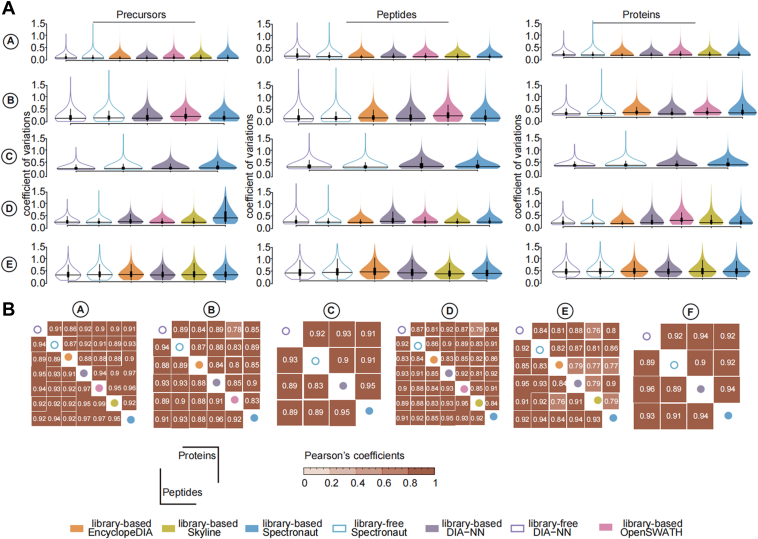
**Evaluation of DIA quantification.***A*, the CVs are calculated at the precursor, peptide, and protein level, and are here plotted for the DIA data analysis tools evaluated in this study. The median values were indicated as text below the violins. *B*, Pearson’s correlations of the peptide and protein intensity were derived from each tool pair. DIA, data-independent acquisition.

In order to assess the accuracy of each tool, we calculated the CVs for precursors, peptides, and proteins. The CV is a measure of variability, with lower values indicating higher accuracy and consistency. We observed that the CVs were generally lowest for proteins, indicating greater stability in their quantification across the different tools. On the other hand, precursors exhibited higher variability, suggesting that their quantification results were more prone to fluctuations. For the datasets obtained from TripleTOF and TimsTOF mass spectrometers, most of the tools achieved a mean CV of less than 10%, indicating relatively good accuracy and consistency. However, in the case of the library-based Spectronaut searches on dataset D, the CVs at the precursor level were higher, exceeding the 10% threshold. In comparison, the Orbitrap datasets exhibited higher CVs than the datasets obtained from other types of mass spectrometers. This was particularly evident at the precursor level, where the mean CVs were below 30%. These findings provide insights into the accuracy and variability of the quantification results obtained by each tool across different types of mass spectrometers and datasets (Fig. 5*A*).

Next, we evaluated the cross-tool quantification efficiency using pairwise correlation between the intensity of peptides and proteins computed through different tools (Fig. 5*B*). The Pearson’s coefficients between different tools were all greater than 0.75, indicating that all the DIA tools provide a relatively high-quantification consistency. The correlation coefficients between peptide intensity were higher than those of protein intensity for the Orbitrap and TripleTOF datasets, whereas they were comparable for the TimsTOF Pro datasets. In addition, we compared the RT difference by computing the pairwise correlation coefficients of the chromatographic peak apexes (supplemental Fig. S10). We found the RT and the peptide intensity correlation coefficients followed similar trends: the more robust the correlation in the RTs, the stronger the correlation in the intensity. Also, we found minor RT difference (median = 0), and the RT correlation coefficients were generally high (>0.98). Indeed, the RT correlations were higher than those for the peptide and protein intensity. These results show that similar peaks were picked, although they may derive from distinct transitions or varying peak boundaries that may result in different integrative values used to represent the abundance of peptides and proteins. This suggests a high consistency of all the examined tools when performing peak picking.

The quantification accuracy was further evaluated using the known true ratios from the LFQ datasets A, B, and C (Fig. 6). Our results showed relatively high accuracy for most samples where the median of the species ratios was closest to the ratios. The quantification accuracy of proteins was better than that of peptides in all three LFQ datasets. The performance of library-free quantification for the larger mixed ratios from TripleTOF (*e.g.,* 1:4 for *E. coli* in dataset A) was slightly worse for peptides, but were relatively stable for the Orbitrap datasets.

### Performance Evaluation Using a Publicly Available Library and Hybrid Library Searches

The above studies compared library-free analysis with library-based analysis with the spectral library created from the dataset within this study. Next, we included some public libraries prebuilt using multiple types of tissues or cell lines, namely the DIA pan-human library version 2 (DIA pan-human library version2, DPHL v2) (26). In addition, some tools like Spectronaut and DIA-NN can provide the option for hybrid search approach, which uses a predicted library in combination with the project-specific deep library.

Here, we compared the performance of multiple library strategies, including the project-specific library, library-free searches from our current results, DPHL v2, and a hybrid library generated from predicted library-free searches in combination with project-specific library by DIA-NN. We tested on the Orbitrap-testis dataset E, which has the largest spectral library with protein isoforms, and adopted DIA-NN, which outperformed other tools in the analyses described above (supplemental Fig. S11).

The hybrid library-search strategy led to the highest number of identifications for both peptides and proteins, although the difference compared to the project-specific library was marginal. The publicly available DPHL v2 library achieved a similar level of identifications as the project-specific library in terms of peptides and unique proteins, but it was still lower than the other two methods. Concerning protein isoforms, library-free searches identified a greater number of protein groups, potentially due to the multiplicity effect in protein grouping. DPHL v2 was able to identify proteins absent in the tissue-specific libraries but further validation is necessary to confirm its presence.

### A Web Server for Unified Format and Comparison

To standardize the representations of precursors, peptides, and proteins reported by different DIA tools, we created an R-based platform. This platform takes the output matrices generated by each of the five DIA tools as input and produces unified precursor, peptide, and protein expression matrices. These matrices can then be used for further comparative analyses, enabling consistent and streamlined data interpretation across different tools. A freely accessible web server was assembled to unify the search results generated by different DIA data analysis tools, providing a user-friendly comparison platform: https://www.guomics.com/softw/diatoolcomp. Also, the codes in converting search results and data analysis are available at https://github.com/guomics-lab/DIAToolComp.

## Discussion

Although a first evaluation of the available data analysis tools was performed a few years ago (19) when DIA-based proteomics regained its popularity, it only included TripleTOF datasets and a few tools. During the past few years, these tools have been substantially improved. In our work, we compared five DIA data analysis tools (Spectronaut, EncylcopeDIA, DIA-NN, OpenSWATH, and Skyline), using data generated by the three types of mass spectrometers commonly used for DIA (TripleTOF, Orbitrap, and TimsTOF Pro).

A recent work by Gotti *et al.* (20) compared the performance of six tools, including DIA-NN, DIA-Umpire, OpenSWATH, ScaffoldDIA, Skyline, and Spectronaut, but this study has several unique features. Firstly, Gotti *et al* was performed on artificially simplified samples, which only contains 48 human proteins with *E. coli*. proteome as background. These simple samples cannot reflect the real complexity of most proteomic applications. In addition, their analyses were only performed on the Orbitraps-based DIA datasets, whereas in our study we comprehensively compared three types of MS instruments. We compared the latest released versions of each software tools, including DIA-NN (version 1.7.5 from 2021), while Gotti *et al* adopted the 2020/04/20 version of DIA-NN. There have been significant improvements in the performance between these two versions. With the new version, we concluded that DIA-NN is superior in most DIA-MS data analyses. Our work also included more characteristics in terms of performance, including speed comparison and physicochemical properties of cross-tool overlapping entities. Finally, we provided an integrative server for comprehensive analysis of user’s inputs (27).

The specific implementations of these five tools are not identical. EncyclopeDIA scores the non-RT chromatographic features of X!Tandem hyperscore and 15 additional features to be trained using Percolator (28), a semisupervised method support-vector machine-based tool for the first round FDR corrections. Then, EncyclopeDIA uses the most confidently detected peptides to estimate the RT distribution to filter the transitions fitting the peak shape, and it finally quantifies the peptides for another round of FDR correction with Percolator. OpenSWATH performs the RT alignment against the RT calibration peptides and then extracts the ion chromatograph. The extracted peak groups are scored using orthogonal features in PyProphet (29), a semi-supervised linear discriminative analysis–based learning algorithm originally applied in analyzing the selected reaction monitoring data. DIA-NN (11) performs the RT calibration of the endogenous peptides. It then uses 73 liquid chromatography and MS scores as the input in training a deep neural network–based model to obtain discriminant scores for FDR estimation. Skyline-targeted analysis first extracts peak groups with seven significant scores and then performs automatic peak picking using mProphet or other algorithms. Lastly, the commercial software Spectronaut uses a core algorithm originally adopted from selected reaction monitoring and mProphet-based algorithms. DIA-NN and Spectronaut were provided with library-free search options but their principles in spectral library-free searches are different. The module directDIA in Spectronaut utilizes the similar principle by DIA-Umpire, which firstly generates DDA-like pseudospectra and then performs searches. Whereas DIA-NN’s library-free search uses computational spectral library generated from the provided sequence database and then performs a DIA style targeted/peptide-centric analysis.

In our evaluation, we observed that despite the variations in implementation and adjustable parameters among the DIA data analysis tools, the results they produced were highly consistent. This consistency was particularly evident in the identification and quantification of proteins, where a significant portion of the proteins identified were consistently identified across all tools. Furthermore, when comparing the quantification results between different tools, we found robust correlations with Pearson's coefficients exceeding 0.75. These consistent results across multiple datasets demonstrate the robustness of DIA-based quantitative proteomics and highlight the reliability of the analyzed tools.

From our comparative results, DIA-NN demonstrated the best performance in terms of identification, computational efficiency, and compatibility when analyzing different formats of DIA-MS data. DIA-NN comes with either a simple graphic user-interface version or a command-line executable version running on Linux clusters. More importantly, it is open-source software. The developers actively respond on the DIA-NN issue forum, and it is currently frequently updated. It also supports multiple operating systems. However, its noncommercial and academic nature hinders its productivity in translating to the biomedical industry, which requires a complete customer support service. It is noteworthy that DIA-NN is no longer an open-source tool since version 1.7.12. The source codes for DIA-NN have been eliminated from its GitHub repository. In contrast, commercial software Spectronaut, which supports different DIA data formats and provides tolerable computing time, is a valid alternative. In particular, Spectronaut encompasses a variety of downstream analysis pipelines that can help inexperienced users. The remaining three tools tested by this study did not support different search modes or data formats in our evaluation.

The development of deep learning–based approaches for library prediction has sparked discussions about the necessity of building spectral libraries for DIA analyses. Our study investigated this matter and found that while library-free methods can outperform library-based methods when the library is very small (*e.g.,* containing only 3000 proteins for human encoding proteins), they showed lower sensitivity and poorer RT concordance than library-based methods when larger libraries are used. This indicates that building a comprehensive library is still crucial for most DIA analyses. Additionally, the width of the DIA isolation window is another important factor that can influence the performance of library-free searches. Interestingly, we observed that datasets with narrower isolation windows exhibited superior performance in library-free searches. These findings align with previous research by *Navarro et al.* (19), which reported smaller differences in identifications between library-free and library-based approaches when narrow DIA isolation windows were employed. We found that the peptides that were identified by several tools were the most intense ones, whereas the less identified ones had lower intensity and eluted late in the gradient. Furthermore, we observed inconsistency among the low-abundant proteins. This suggests that future software should find ways to address the identification of low-abundant peptides. In addition, other long-standing issues in the DIA data interpretation remain to be improved. These include, but not limited to, identification and confirmation of low abundance, noncanonical, or modified peptides, the precise estimation of peptide and protein FDR, dealing with technical missing values in large datasets, and integrating results derived from different search engines.

It is important to acknowledge that a comparative study of software tools in the field of DIA analysis has inherent limitations. One of these limitations is the inability of providing an up-to-date comparison, as software tools are continually evolving to improve their performance. The comparison provided in our study represents a "state-of-the-art" assessment at the time of analysis, based on specific software versions. Another limitation is the small sample size of the test data used in the study. While efforts were made to select representative datasets, it is recognized that these datasets may not fully capture the diversity and complexity of all DIA-based proteomic datasets. In particular, very large datasets were not included in the analysis. Additionally, the choice to focus on datasets with fairly homogeneous samples was made to minimize variance from sample heterogeneity. However, real-world samples are often more heterogeneous and complex, such as clinical samples from large cohorts. The performance of DIA tools in such complicated samples would require further investigation in future studies. It is important to interpret the findings of our study within these limitations and consider the evolving nature of the field when assessing the performance of DIA analysis software tools.

## Data Availability

All the codes associated with this study including tool conversion scripts for the web server and plotting scripts for data analysis were publicly available at https://github.com/guomics-lab/DIAToolComp. The in-house generated MS dataset and libraries were deposited at PRIDE with the accession PXD039759.

## Supplemental data

This article contains supplemental data.

## Conflict of interest

T. G. is shareholder of Westlake Omics, Inc. W. G., L. H., D. L., and L. L. were employees of Westlake Omics, Inc. when they participated in this project.
