# Supplementary material for: A Comparative Analysis of Data Analysis Tools for Data-Independent Acquisition Mass Spectrometry
Source: Mol Cell Proteomics. 2023 Jul 21;22(9):100623. doi: 10.1016/j.mcpro.2023.100623 (PMC10458344; doi:10.1016/j.mcpro.2023.100623)
Supplement: Supplementary data [file mmc1.docx]

**Supplementary Materials**


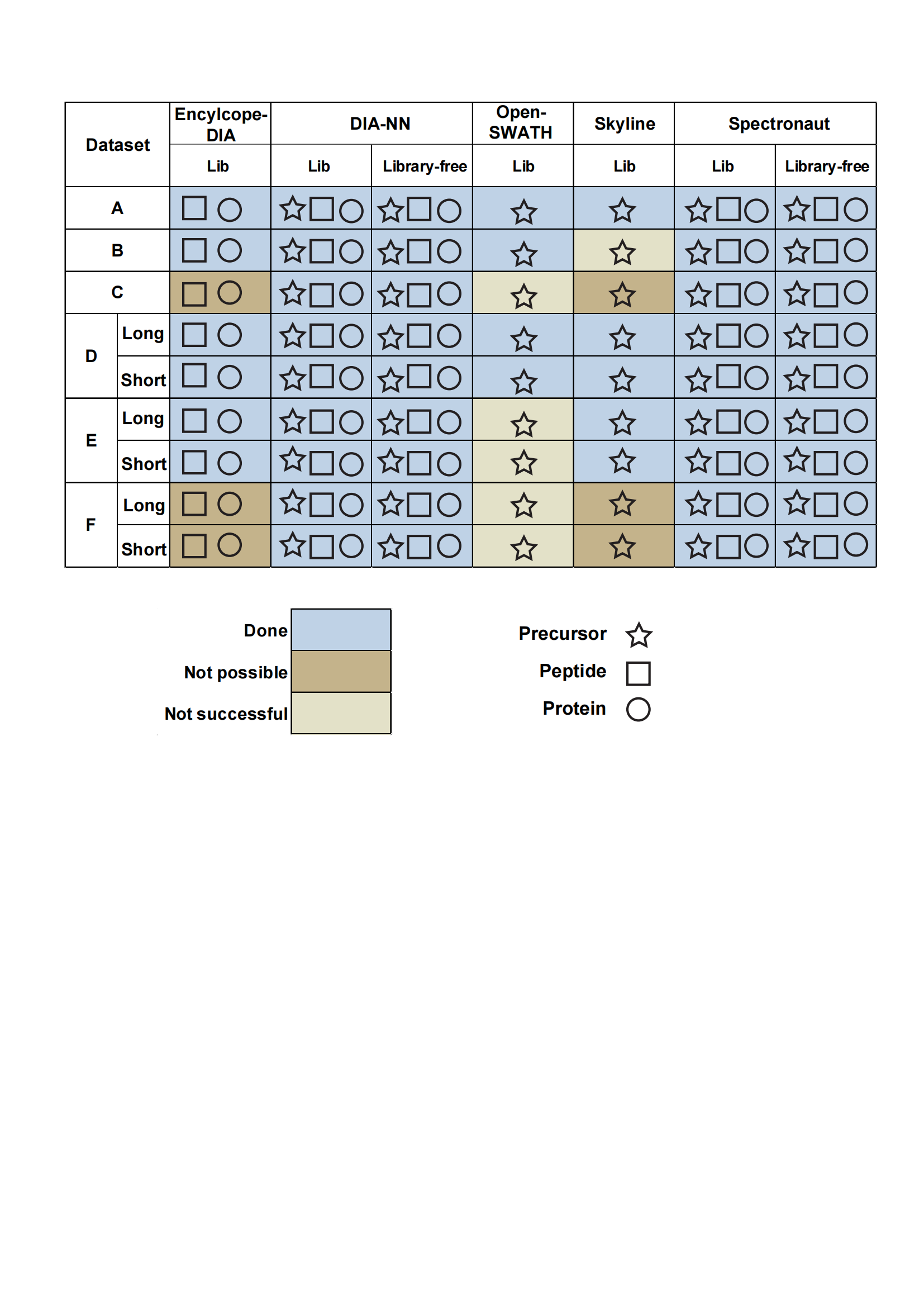
**Supplementary Figure 1. Feasibility of running DIA analyses and final report formats.** For the five tested data analysis tools, with library-based and library-free modes, 63 searches were performed on six datasets. Specifically, 51 search runs were successful (light blue), six were not possible as the software may lack certain required functionalities data (orange). These impossibilities include in processing TimsTOF data for EncyclopeDIA and Skyline. Six were not successful (yellow) due to software technical fatal issues. These includes the failure of running OpenSWATH for TimsTOF datasets and failure of running long gradient samples (2-hour and 6-hour samples) and failure of running Skyline due to the limited separation of target-decoy. The final search reports provided by different tools indicated: precursor matrices (star), peptide matrices (rectangle), protein matrices (circle).

**
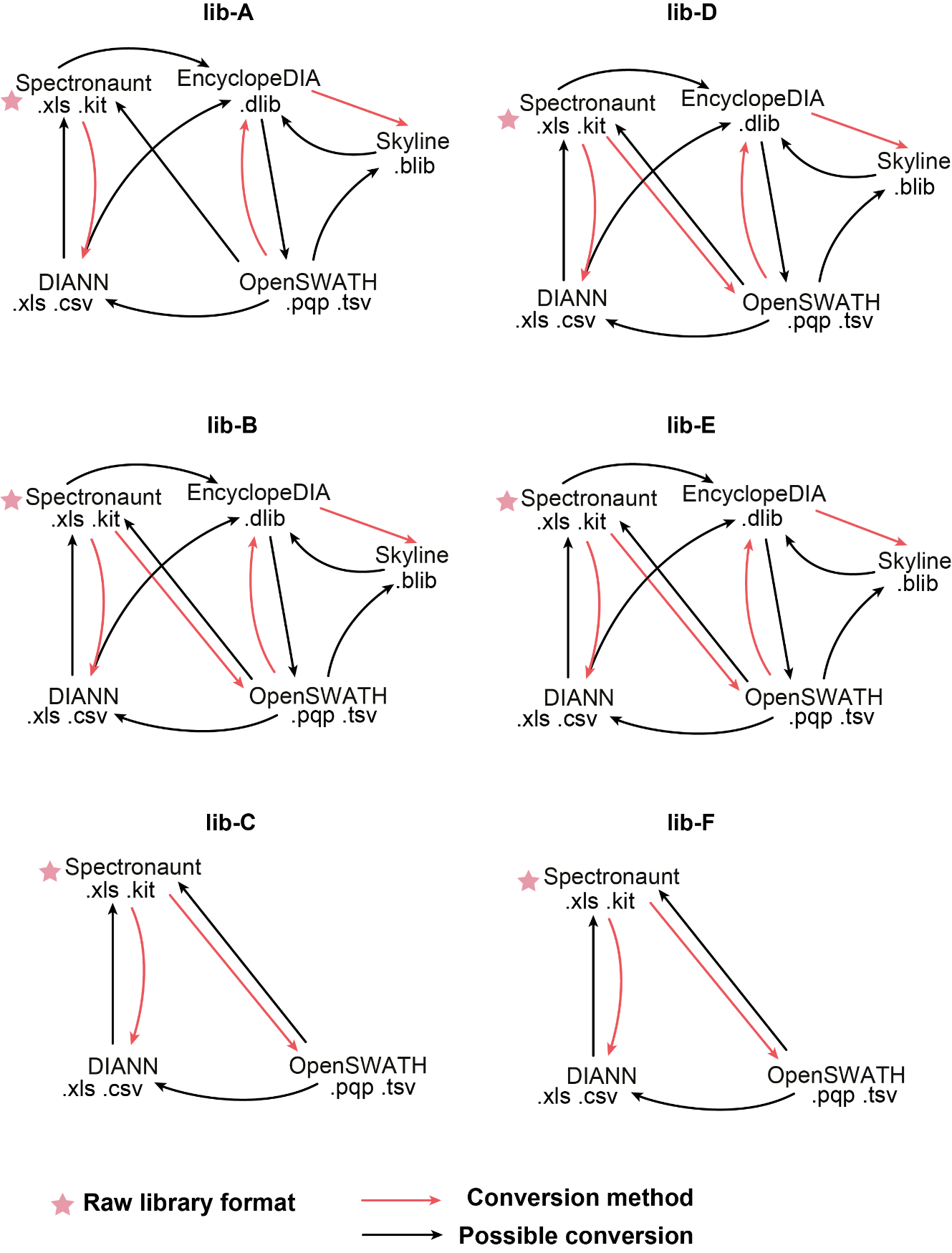
 Supplementary Figure 2. Library conversion scheme.** Each dataset was derived using a different raw library format (stars) from the one used in the original publications as it required a format conversion to be run in each tool. The conversion methods are indicated with red arrows.

**
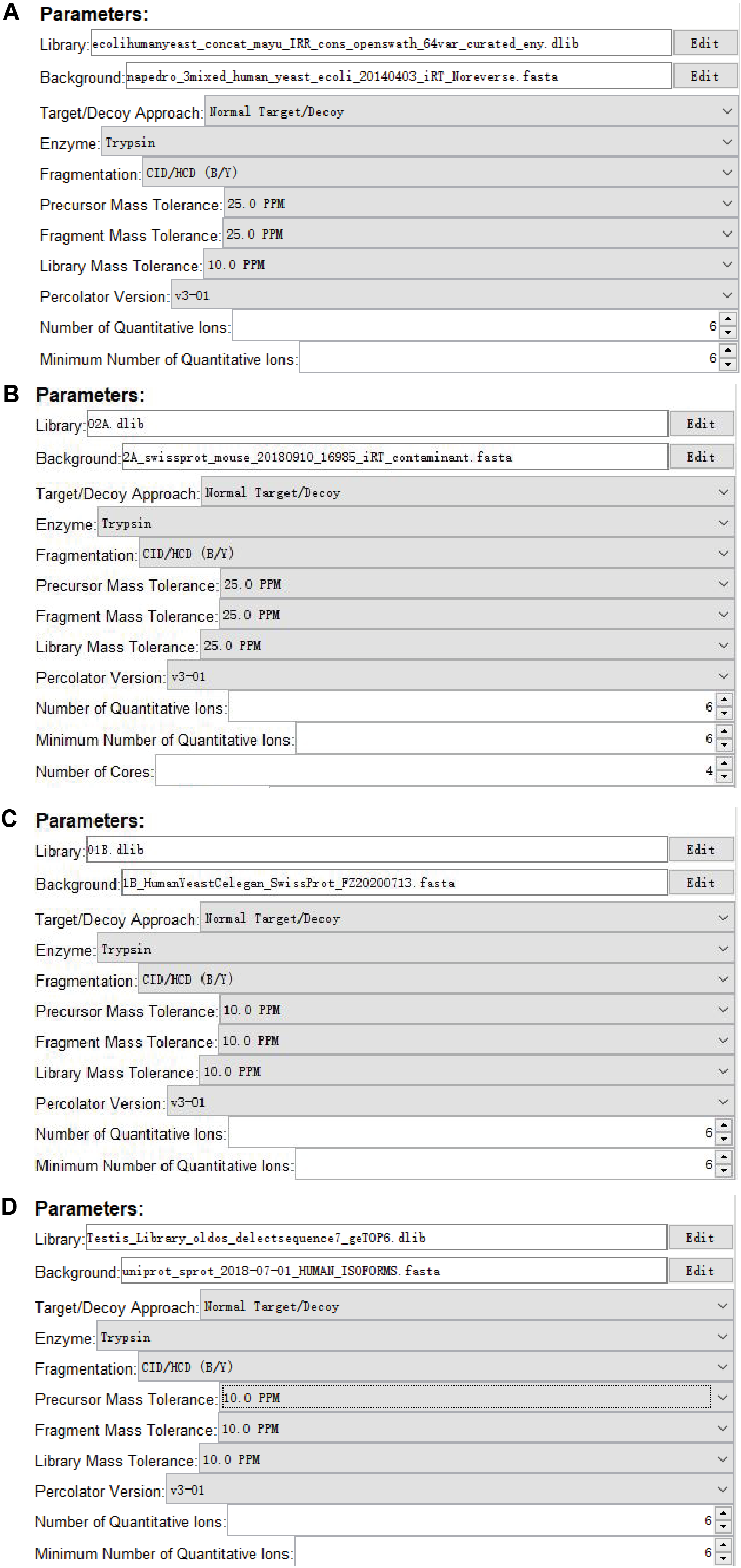
**

**Supplementary Figure 3. Data analysis parameters for EncyclopeDIA.** Screen captures of the input parameters used during the analysis of the six datasets. **A**. Dataset A. **B**. Dataset B. **C**. Dataset D. **D.** Dataset E.


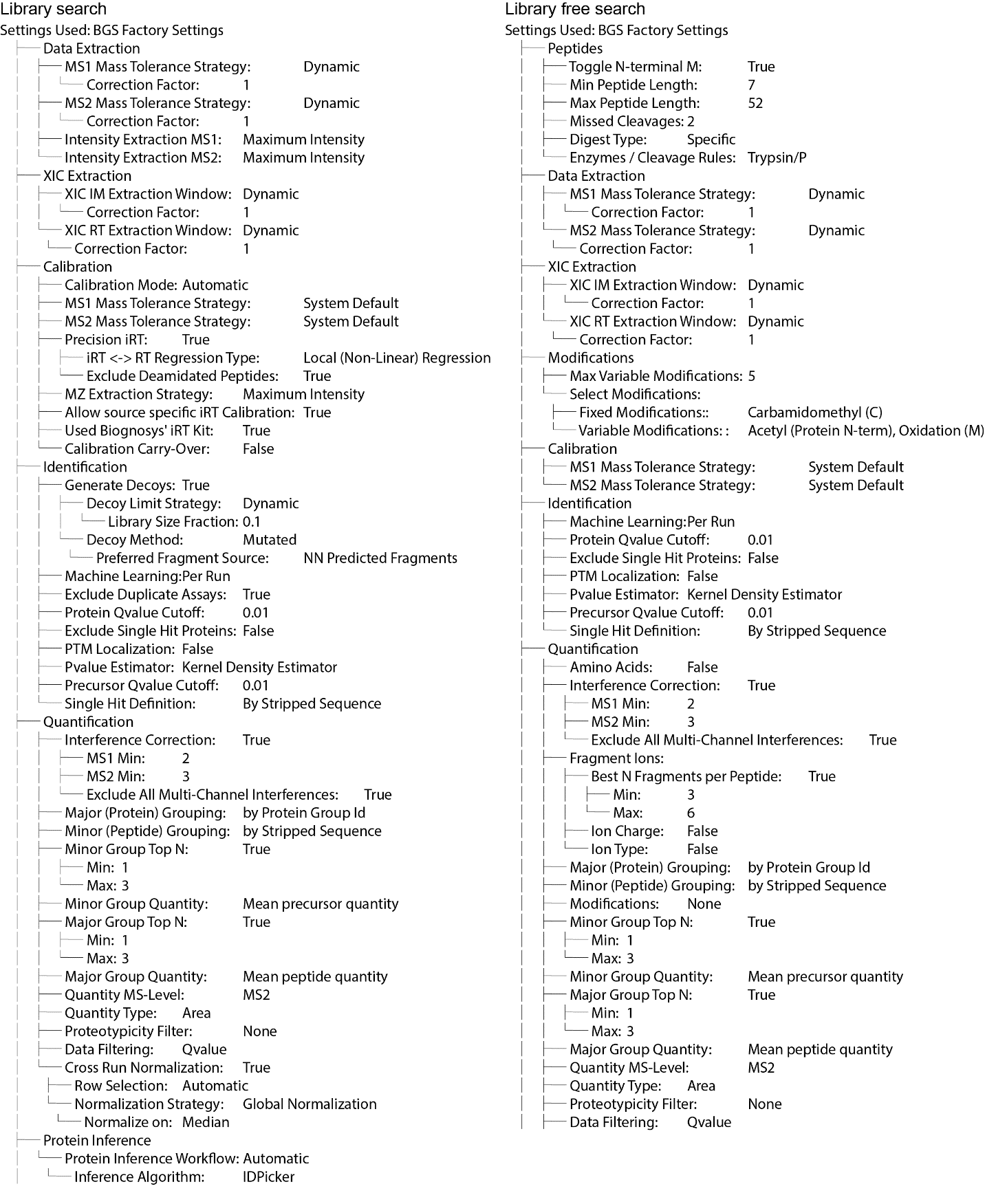


**Supplementary Figure 4. Data analysis parameters for Spectronaut.** All analyses were performed using factory settings without any modifications. Two sample captures from the log file of a library-based and a library-free search are shown here.

**
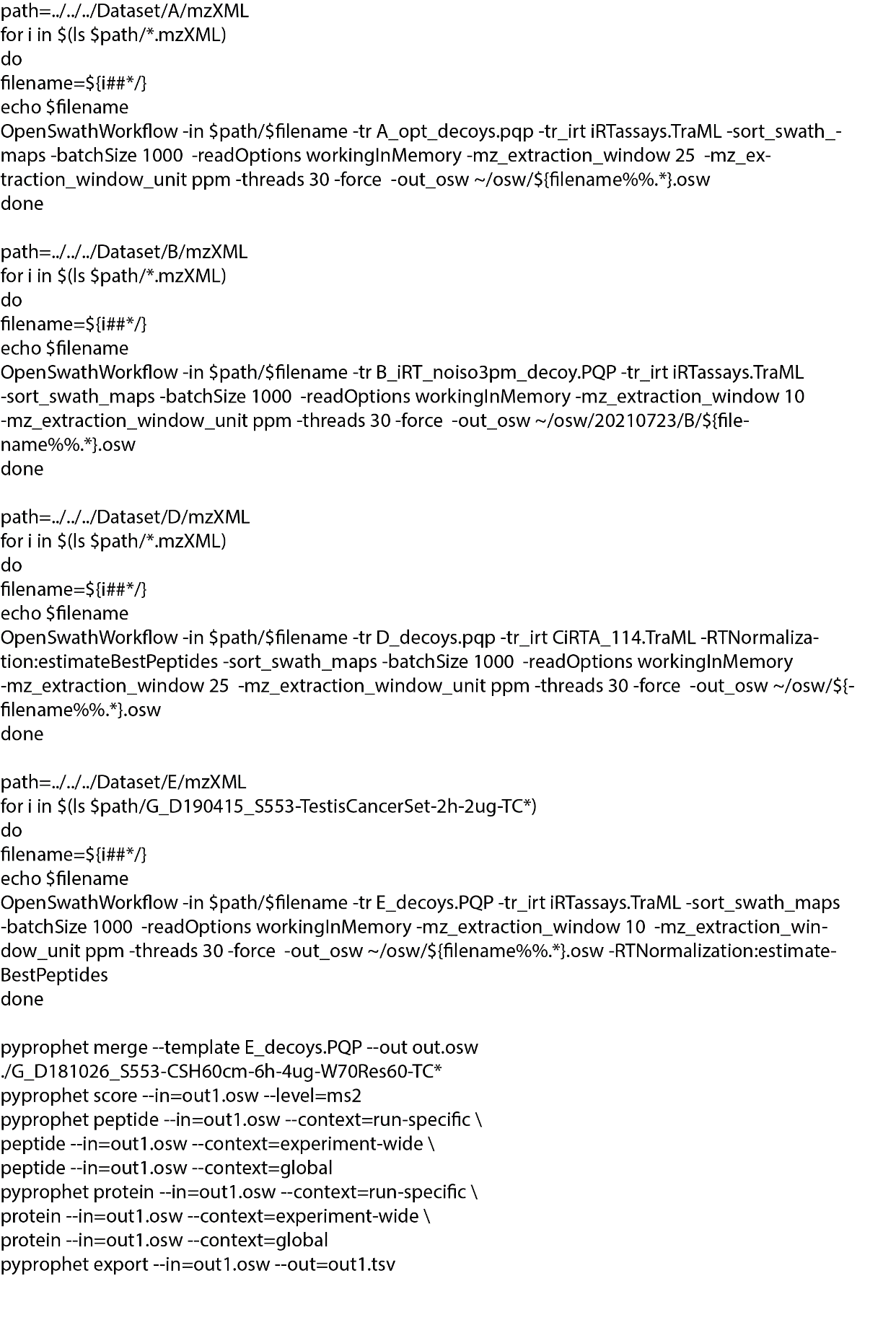
 Supplementary Figure 5. Data analysis parameters for OpenSWATH.** Command-line used for each OpenSWATH run and PyProphet scoring.

#
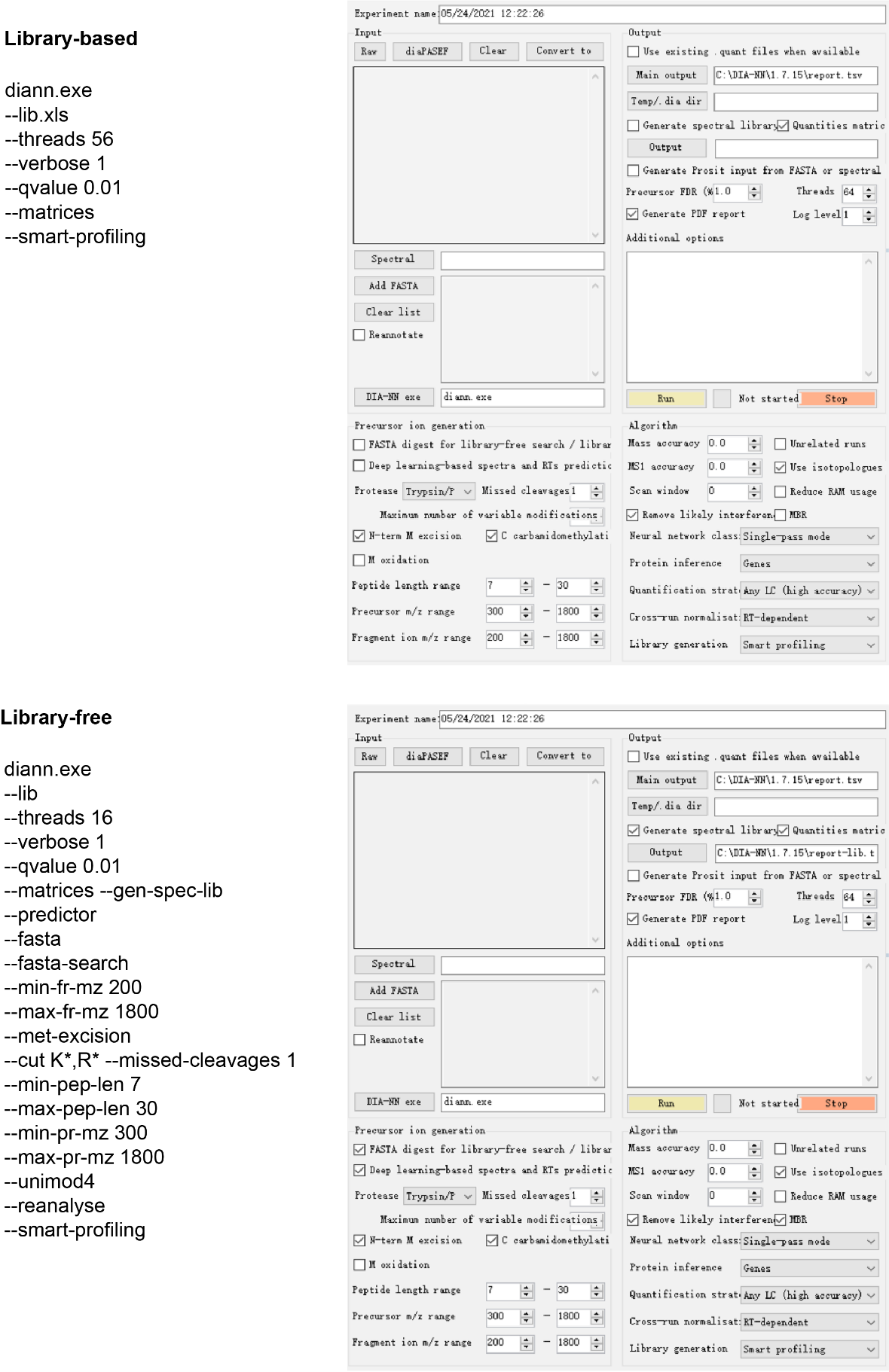


**Supplementary Figure 6. Data analysis parameters for DIA-NN.** All searches were performed using default settings. Here, two sample command lines and graphic user interfaces are provided for library and library-free searches.

**
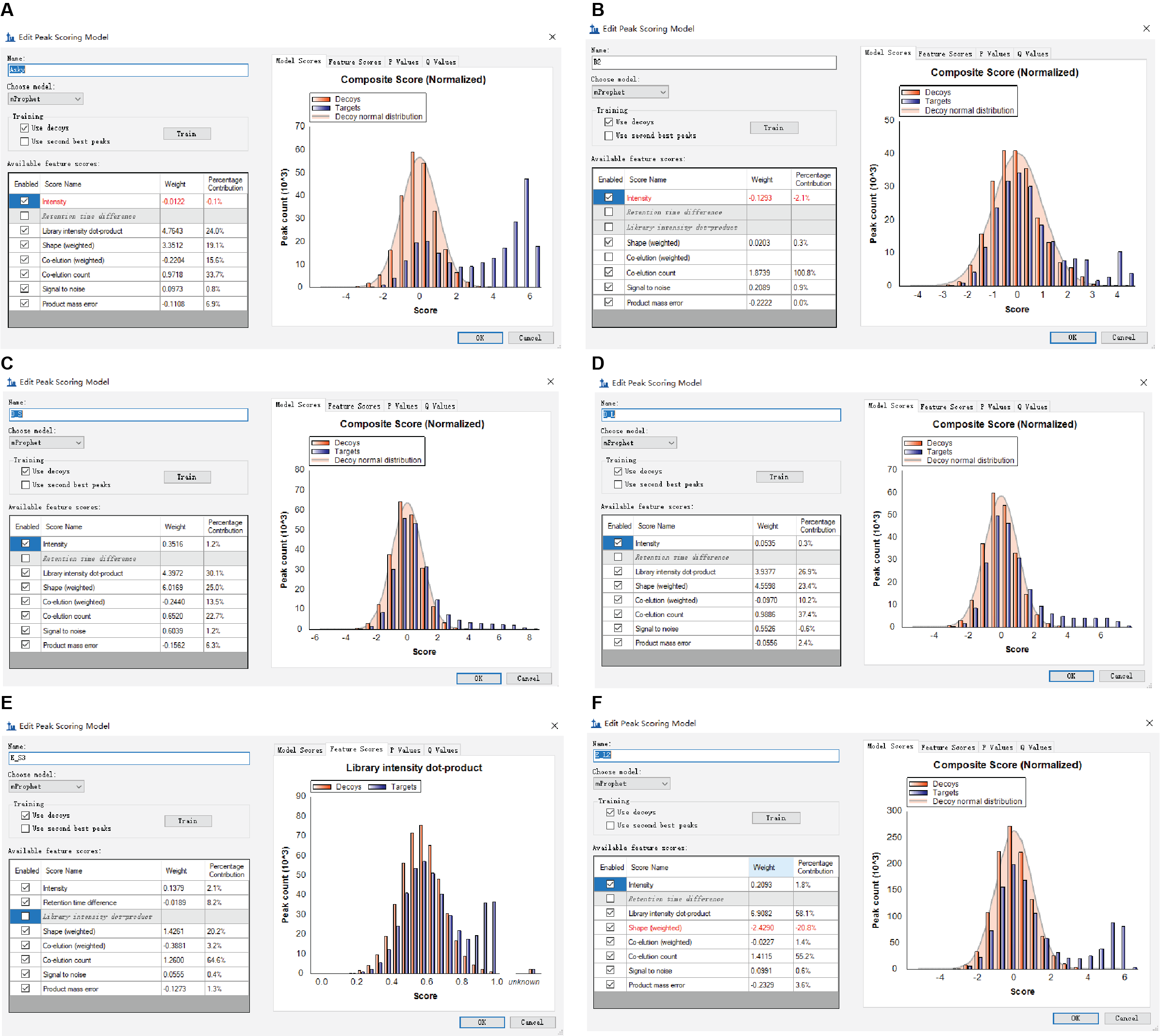
Supplementary Figure 7. Results for Skyline target-decoy separation. A.** Dataset A. **B.** Dataset B. **C.** Dataset D for short gradient**. D.** Dataset D for long gradient. **E**. Dataset E for short gradient. **F.** Dataset E for long gradient.

| **Data ID** | **library search** | **Gradient** | | **Tool** | **CPU specifics** | **Duration of computing time** | **Estimated CPU hours (by #Cores x Duration)** |
| --- | --- | --- | --- | --- | --- | --- | --- |
| A | library-free |  | DIA-NN | | 8280M CPU @ 2.60GHz | 6h23min | 102 |
| A | library-based |  | EncyclopeDIA | | 5122 CPU @ 3.60GHz | 6h46min | 27 |
| A | library-based |  | DIA-NN | | 8280M CPU @ 2.60GHz | 22min | 21 |
| A | library-based |  | OpenSWATH | | E5-2630 v4 @ 2.20GHz | 25h5min | 753 |
| A | library-based |  | Skyline | | 7502 @ 2.5GHz | 7h8min |  |
| B | library-free |  | DIA-NN | | 8280M CPU @ 2.60GHz | 5h20min | 85 |
| B | library-free |  | Spectronaut | | 8280M CPU @ 2.60GHz | 12h27min | 797 |
| B | library-based |  | EncyclopeDIA | | 8280M CPU @ 2.60GHz | 6h10min | 25 |
| B | library-based |  | DIA-NN | | 8280M CPU @ 2.60GHz | 20min | 5 |
| B | library-based |  | OpenSWATH | | E5-2630 v4 @ 2.20GHz | 2h3min | 62 |
| B | library-based |  | Skyline | | 8280M CPU @ 2.60GHz | 11h30min |  |
| B | library-based |  | Spectronaut | | 8280M CPU @ 2.60GHz | 1h49min | 116 |
| C | library-free |  | DIA-NN | | 5122 CPU @ 3.60GHz | 71h7min | 427 |
| C | library-free |  | Spectronaut | | 8280M CPU @ 2.60GHz | 30h48min | 1971 |
| C | library-based |  | DIA-NN | | 8280M CPU @ 2.60GHz | 10h15min | 328 |
| C | library-based |  | Spectronaut | | 8280M CPU @ 2.60GHz | 7h23min | 473 |
| D | library-free | long | DIA-NN | | 8280M CPU @ 2.60GHz | 8h29min | 136 |
| D | library-free | short | DIA-NN | | 8280M CPU @ 2.60GHz | 2h52min | 46 |
| D | library-based | long | EncyclopeDIA | | 5122 CPU @ 3.60GHz | 7h35min | 30 |
| D | library-based | long | DIA-NN | | 8280M CPU @ 2.60GHz | 31min | 8 |
| D | library-based | long | OpenSWATH | | 6148 CPU @ 2.40GHz | 29h26min | 883 |
| D | library-based | long | Skyline | | 7502 @ 2.5GHz | 21h52min |  |
| D | library-based | long | Spectronaut | | 8280M CPU @ 2.60GHz | 6h19min | 404 |
| D | library-based | short | EncyclopeDIA | | 8280M CPU @ 2.60GHz | 2h50min | 11 |
| D | library-based | short | DIA-NN | | 8280M CPU @ 2.60GHz | 7min | 2 |
| D | library-based | short | OpenSWATH | | 6148 CPU @ 2.40GHz | 11h20min | 340 |
| D | library-based | short | Skyline | | 7502 @ 2.5GHz | 18h50min |  |
| D | library-based | short | Spectronaut | | 8280M CPU @ 2.60GHz | 3h | 192 |
| E | library-free | long | DIA-NN | | 8280M CPU @ 2.60GHz | 6h29min | 104 |
| E | library-free | long | Spectronaut | | 8280M CPU @ 2.60GHz | 15h4min | 964 |
| E | library-free | short | DIA-NN | | 8280M CPU @ 2.60GHz | 3h37min | 58 |
| E | library-free | short | Spectronaut | | 8280M CPU @ 2.60GHz | 9h1min | 577 |
| E | library-based | long | EncyclopeDIA | | 8280M CPU @ 2.60GHz | 9h40min | 39 |
| E | library-based | long | DIA-NN | | 8280M CPU @ 2.60GHz | 51min | 14 |
| E | library-based | long | OpenSWATH | | E5-2630 v4 @ 2.20GHz | 22h47min | 684 |
| E | library-based | long | Skyline | | 7502 @ 2.5GHz | 20h45min |  |
| E | library-based | short | EncyclopeDIA | | 5122 CPU @ 3.60GHz | 5h43min | 23 |
| E | library-based | short | DIA-NN | | 8280M CPU @ 2.60GHz | 35min | 9 |
| E | library-based | short | OpenSWATH | | E5-2630 v4 @ 2.20GHz | 1h28min | 44 |
| E | library-based | short | Skyline | | 7502 @ 2.5GHz | 3h9min |  |
| E | library-based | short | Spectronaut | | 8280M CPU @ 2.60GHz | 7h29min | 479 |
| E | library-based | short | Spectronaut | | 8280M CPU @ 2.60GHz | 3h22min | 215 |
| F | library-free | long | DIA-NN | | 5122 CPU @ 3.60GHz | 33h39min | 34 |
| F | library-free | short | Spectronaut | | 8280M CPU @ 2.60GHz | 8h29min | 543 |
| F | library-free | short | DIA-NN | | 5122 CPU @ 3.60GHz | 33h13min | 33 |
| F | library-based | long | Spectronaut | | 8280M CPU @ 2.60GHz | 9h39min | 618 |
| F | library-based | short | Spectronaut | | 8280M CPU @ 2.60GHz | 3h20min | 213 |

**Supplementary Figure 8. Computing specifics and time consumption of each data analysis tool.** The number of cores, the CPU specifics, and the computing time are here provided.


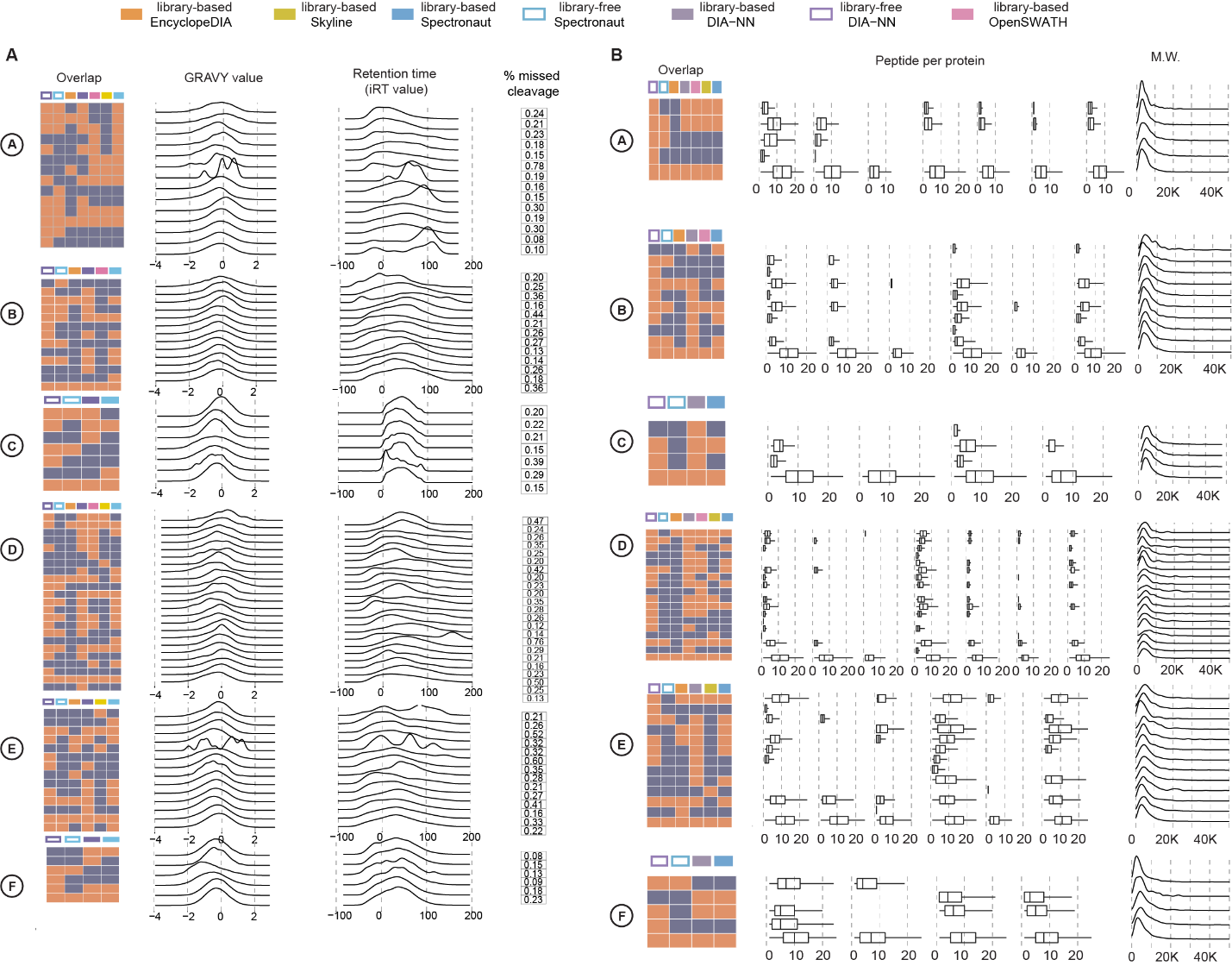


**Supplementary Figure 9. Extended characterizations of peptide and protein properties. A.** For peptides, further properties include retention time in the library (if any), the hydrophobicity GRAVY value, and the missed cleavages ratios. **B**. For proteins, peptides per proteins for all searching tools. The distribution of molecular weights is also shown.

#
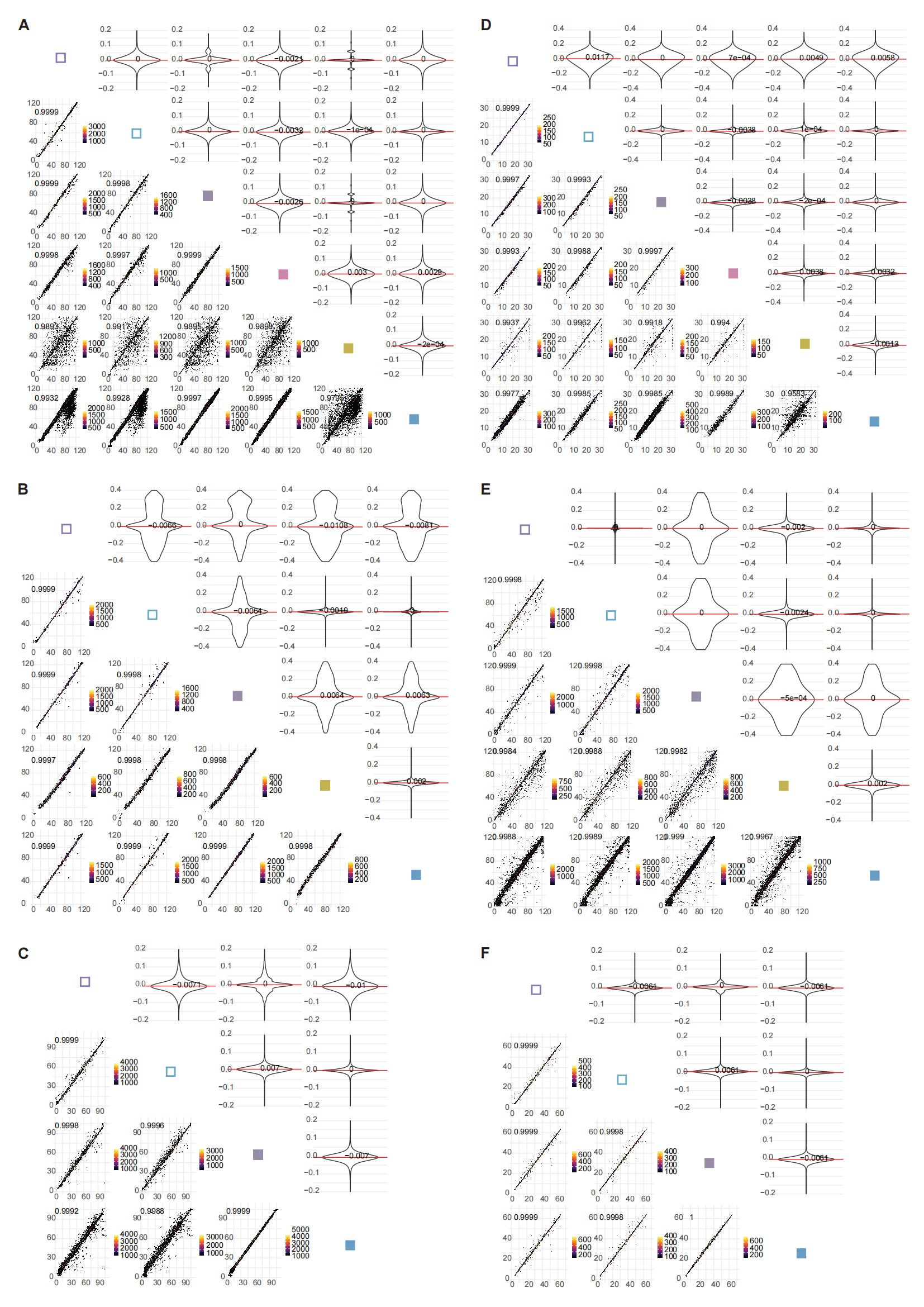


**Supplementary Figure 10. Distribution of the cross-tool retention times.** The upper right triangles show the violin plots of the RT differences of pairwise matching peptide precursors. The lower left triangle panels show the pairwise Pearson’s correlation plots from match peptides. The data analysis tools and the library searching methods are indicated in the middle diagonals (same color code used for Figure 3).


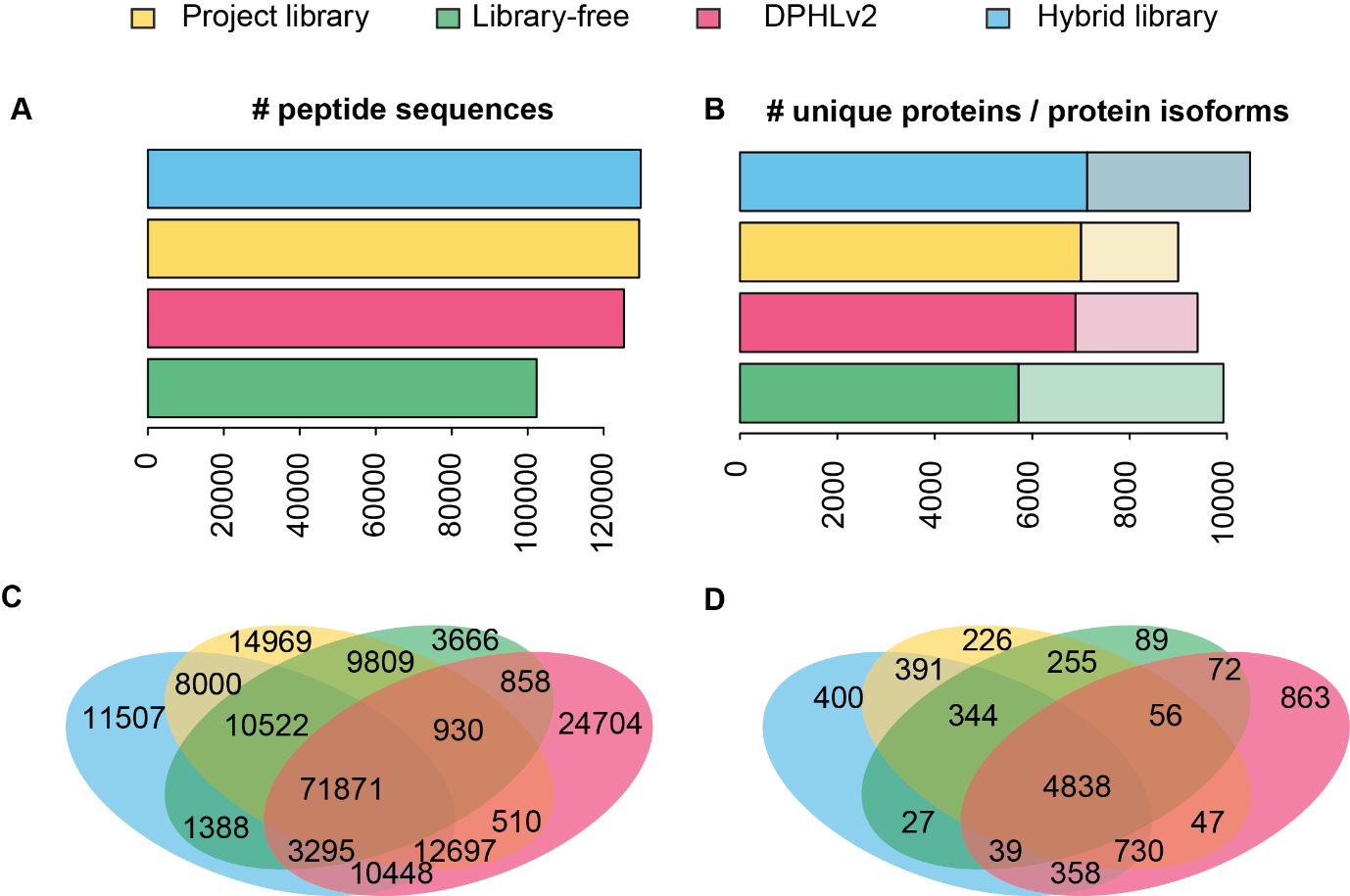


**Supplementary Figure 11. Comparison of the performance of different libraries using DIA-NN.** Four searching methods were tested, including a project specific library derived from original study (yellow); library-free searches with fasta sequence library (green); a public library named DIA orbitrap human library version 2 (DPHL v2) (red); a hybrid library by combining the project specific library with predicted library from library-free search results (blue). (A) Number of identified peptide sequences. (B) Number of unique proteins and protein isoforms (in shade). (C) Overlaps of identified peptides. (D) Overlaps of identified unique proteins.
